# Stakeholder perceptions of physical literacy: results from a national consultation in England

**DOI:** 10.3389/fspor.2024.1457845

**Published:** 2024-10-17

**Authors:** Michael J. Duncan, Inimfon Essiet, Liezel Hurter, William M. Roberts, Kiara Lewis, Hannah Goss, Jade L. Morris, Daniel D. Bingham, Wesley O’Brien, Lisa M. Barnett, Cara Shearer, Andrew Daly-Smith, Lawrence Foweather

**Affiliations:** ^1^ Centre for Physical Activity, Sport and Exercise Science, Coventry University, Coventry, United Kingdom; ^2^ Research Institute for Sport and Exercise Sciences, Liverpool John Moores University, Liverpool, United Kingdom; ^3^ School of Sport and Human Movement, University of Waikato, Hamilton, New Zealand; ^4^ School of Health Sciences, Birmingham City University, Birmingham, United Kingdom; ^5^ School of Health and Human Performance, Dublin City University, Dublin, Ireland; ^6^ Faculty of Health Studies, University of Bradford, Bradford, United Kingdom; ^7^ School of Education, University College Cork, Cork, Ireland; ^8^ Institute of Physical Activity and Nutrition, Deakin University, Melbourne, VIC, Australia; ^9^ Sport Scotland, Glasgow, United Kingdom

**Keywords:** active lifestyles, competence, confidence, motivation, physical activity, knowledge and understanding

## Abstract

**Background:**

There is a lack of evidence of stakeholder perspective and understanding of physical literacy among relevant stakeholders from England. As part of research commissioned by Sport England to develop a physical literacy consensus statement for England, this study presents findings from the first national consultation with stakeholders in England.

**Methods:**

One hundred and ninety-three individual stakeholders (50.3%) from education, community sport, national governing bodies of sport, physical activity and sport coaching sectors completed an online survey consisting of fixed item and open ended questions designed to examine their knowledge, understanding, perceptions and practices relating to physical literacy.

**Results:**

Responses from stakeholders suggested there was confusion in use of the term physical literacy in practice and confusion regarding the definition of physical literacy. Most respondents suggested they were involved in physical literacy related activity and understood the term. However, when probed the physical literacy related activity they referred to was likely not actually physical literacy related. Understanding of the term physical literacy was inconsistent in general. Stakeholders considered the affective, social, physical and cognitive areas (domains) of learning to be most important for developing a positive relationship with movement, sport and physical activity for life.

**Conclusions:**

While stakeholders are aware of the term “physical literacy” and hold value of it within their practice, there remain key misconceptions relating to what physical literacy is, and debate as to whether any existing definitions truly capture the construct of physical literacy.

## 1 Introduction

Physical literacy has gained widespread and worldwide attention in the education, health, sports, recreation and public health sectors a framework for nurturing sustained engagement in physical activity across the life course (1–4). The term “Physical Literacy” is not new and has existed since the late 19th century, finding its earliest use in describing the movement quality/physicality of an indigenous American population (5). Whitehead reimagined the concept, refining her definition of physical literacy over the years, with the most recent being “the motivation, confidence, physical competence, knowledge and understanding to value and take responsibility for engaging in physical activities for life” (2). Essentially, the holistic development of certain core competencies, skills, and attributes increases an individual's likelihood of sustained and lifelong engagement in physical activity. Recent research has noted the widespread interest in physical literacy in terms of research, policy and practice (3). Whilst there are differences in concept, measurement of, understanding, definition and philosophical underpinnings, there is general agreement that physical literacy is a lifelong concept, that should be inclusive in nature, and includes interacting domains that are physical, cognitive and affective (6). Importantly, physical literacy is a term that features in national and international policy guidelines such as those produced by the World Health Organization (7), and in UNESCO's quality physical education policy (8). Yet to date, we have very little evidence for what stakeholders in England understand physical literacy to be, and also what stakeholders in England state they need in terms of training and support to implement it in practice.

Several research fields in the sport, exercise, and health sciences (9) are increasingly advocating for research that includes stakeholders. The notion of “nothing about us without us” is prevalent and perhaps those researching in physical literacy should consider how to better co-design research to reach consensus that moves beyond the philosophical, to the pragmatic and practical, in line with recommendations for research in the sport, exercise and health sciences in general (9).

Despite the interest in physical literacy as a concept (4, 5, 10), there remains a lack of consensus on the definition of physical literacy, its constituting components/elements, and underpinning philosophical tenets. Hurter et al.'s (10) recent evidence review identified 23 different definitions designed by a range of authors, organisations/countries, compounding the issues stakeholders will likely face in implementing key concepts. The framing of these definitions is often shaped by the cultural significance of the concept in the countries they are adopted and/or organisations' specific purposes and areas of expertise (1, 11, 12). Young et al. (13) recently sought to address the continued controversy and confusion by arguing that physical literacy should be understood as a multiverse of co-existing literacies that play coexisting roles based on the problem that each actor is trying to solve (i.e., health-promoting physical activity, motor competence, or phenomenological embodiment). Whilst tolerance for different approaches for defining and conceptualising physical literacy have been suggested (12–14), the existence of these different definitions generates inconsistency within the field (12, 15) and could result in considerable confusion and lack of understanding by relevant stakeholders (e.g., teachers, coaches, health professionals) interested in operationalising the concept (16). These stakeholders are crucial in supporting individuals on their physical literacy journey through the life course, yet to date there is a lack of research examining stakeholder perspectives and understanding of physical literacy.

Stakeholder evidence from studies among teachers in Australia, Canada, and the United States have repeatedly highlighted partial and misconstrued understandings of the concept (16–20). To the best of our knowledge, only a small number of published studies have considered physical literacy understandings of stakeholders aside from teachers (21–24); with only one of these considering stakeholders from outside the education sector. The Belton et al. (21) study, which sampled coaches, teachers, researchers, sport leaders and service providers in Ireland and Northern Ireland suggested that more than half were aware of the concept. The work by Foulkes et al. (24) and Buckler and Bredin (23) examined the understanding of physical literacy in early childhood educators, where practitioners agreed physical literacy was an important construct but had a limited understanding of what physical literacy constituted, and a need for training to understand better the concept and how to integrate into practice.

If relevant stakeholders such as teachers and coaches cannot clearly articulate the concept, then, in practice will there will be a lack of clarity on how stakeholders can practically support physical literacy development in those they work with. Before developing interventions, designing curricula and pedagogy to support practice and policy actions to enhance physical literacy, it is important to firstly ensure there is a common understanding of physical literacy with the multiple stakeholders that work within and across this concept in a given country and/or context.

There is a lack of evidence of stakeholder perspective and understanding of physical literacy among relevant stakeholders from England. As part of a broader piece of research commissioned by Sport England to develop a physical literacy consensus statement for England, this study presents findings from the first national consultation with stakeholders in England. This study sought to capture current understanding and perceptions of physical literacy among key stakeholders among individuals/organisations in England.

## 2 Materials and methods

### 2.1 Design

This study used an anonymous online survey, designed for stakeholders working in any capacity where physical literacy is relevant, and asked for their practices and perceptions related to physical literacy. The design of the survey (i.e., protocol, structure, items) was based on psychometrically valid guidelines for constructing questionnaires as well as on the methods utilized in other relevant studies with early years professionals (25), sports coaches (26), and teachers (27). The survey was developed with guidance from Sport England in terms of the key aspects of perceptions and practices of physical literacy from the point of stakeholders. Members of the research team, all experts in the broad area of physical literacy contributed to the development of the questionnaire via question development and subsequent member checking. The survey was pilot tested prior to launch with a small (*n* = 10) cohort of stakeholders, who did not participate in the subsequent roll out of the survey. The survey was designed to be completed in approximately 15 min and was created in and administered through the JISC Online Survey administration application and comprised a variety of fixed response, Likert type and open ended (free text) questions. An optional demographics section was included at the end of the survey. The study was approved by Coventry University Ethics Committee.

#### 2.1.1 Participants

To target the relevant population for this study, survey invitations were sent via e-mail to “Sport England” partners and disseminated via social media to relevant groups working or volunteering with young people in areas related to physical literacy (for example, national governing bodies of sports, schools' games officers, community sports providers, coaching organisations). The participants were drawn from 121 organisations who had previously worked with Sport England on projects potentially related to physical literacy. The list of organisations which were targeted can be found here: Long-term partnerships | Sport England. The decision to focus on these groups was taken in consultation with Sport England, as it was considered these groups would be most likely to be involved in operationalisation of the Sport England consensus on physical literacy. Participants were recruited via invitations which included a brief explanation of the study (i.e., its objectives, aims and rationale), and the hyperlink to the survey. All participants provided written informed consent prior to undertaking the survey. A copy of the survey can be found in the Supplementary Material.

### 2.2 Survey results

One hundred and ninety-three individual stakeholders participated and completed the survey, of which 166 participants opted to provide demographic information: 50.3% (*n* = 83) male and 49.7% (*n* = 82) female. Most respondents described their ethnic background as “white” (93.4%, *n* = 155). Nearly two-thirds (62.7%, *n* = 121) were responding in a personal capacity and were drawn from a wide range of stakeholder groups. Although most respondents reported their job role as “other”, when prompted to explain their role, there was considerable diversity in the job roles reported, including “CEO” [Chief Executive Office], “Insight Officer”, “work for an NGB” [National Governing Body of Sport], “Mentor” and “School Games Officer”, amongst others. When asked which sector/s respondents worked in, the majority reported the Education (*n* = 121, 62.7%) and Sport (*n* = 123, 53.7%) sectors, followed by Physical Activity (*n* = 103, 53.4%). In relation to perceived stakeholder competence in physical literacy, over half of respondents self-rated their level of expertise as either “good” (42.5%, *n* = 82) or “excellent” (11.4%, *n* = 22) with only two respondents (1%) suggesting “none”.

#### 2.2.1 Importance of areas of learning and development for physical literacy

Table 1 presents the relative importance of different areas of learning and development in relation to developing a person's positive relationship with movement, sport, and physical activity for life. There was some variability across the domains regarding their relative importance. Although most participants rated all domains of learning and development as “Somewhat Important” or “Extremely Important”, there were marked higher responses for Affective, Physical and Social domains, where the majority of respondents (94.4%, 72% and 73.9% respectively) rated these domains as “Extremely Important”.

**Table 1:** The relative importance (number and percentage) of different areas of learning related to developing physical literacy from the perspective of stakeholders.

	Not important at all	Somewhat unimportant	Neutral	Somewhat important	Extremely important	Don't know
	*n* (%)	*n* (%)	*n* (%)	*n* (%)	*n* (%)	*n* (%)
Affective	1 (0.5)	0 (0)	1 (0.5)	9 (4.7)	182 (94.4)	0 (0)
Cognitive	1 (0.5)	2 (1)	7 (3.6)	82 (42.5)	100 (51.9)	1 (0.5)
Creativity	1 (0.5)	4 (2)	19 (9.8)	80 (41.5)	88 (45.6)	1 (0.5)
Cultural	1 (0.5)	3 (1.5)	18 (9.3)	82 (42.5)	88 (45.6)	1 (0.5)
Language	3 (1.5)	10 (5.2)	38 (19.7)	89 (46.1)	51 (26.4)	2 (1)
Moral	3 (1.5)	8 (4)	21 (10.9)	68 (35.2)	92 (47.7)	1 (0.5)
Physical	0 (0)	4 (2)	4 (2)	42 (21.8)	139 (72)	4 (2)
Sensory	4 (2)	9 (4.7)	34 (17.6)	79 (40.9)	65 (33.7)	2 (1)
Social	1 (0.5)	4 (2)	5 (2.6)	40 (20.7)	141 (73.9)	2 (1)
Spiritual	8 (4.1)	16 (8.3)	58 (30.1)	68 (35.2)	41 (21.2)	2 (1)

Conversely, the Spiritual and Language domains had a greater proportion of respondents who rated these domains as “Neutral” (30.1% for Spiritual and 19.7% for Language), or as “Somewhat Unimportant” (8.3% for Spiritual and 5.2% for Language) or “Not Important at All” (4.1% for Spiritual and 1.5% for Language).

#### 2.2.2 Elements within physical literacy

Stakeholders were asked what they felt was important for developing a positive relationship with movement and physical activity and to rate different elements on a 5-point Likert scale ranging from “Not Important at All” to “Extremely Important” within each component of physical literacy'. The percentage of respondents who ranked each element as either “Somewhat Important” or “Extremely Important” was summed to create a score for combined importance of each element. Scores were graded using a traffic light system where green = 90% importance, amber ≥75% but <90% importance, and red ≤75% importance. The results from this analysis are presented in Table 2. From this, it appears elements within the “Physical Domain” are overall rated as less important, when compared to the elements for other components of physical literacy. Conversely, there were a larger number of elements within the affective component for physical literacy that were ranked as important. Of 37 elements across all domains, 28 were ranked as “Somewhat Important” or “Extremely Important” by 75% or more of stakeholders.

**Table 2:** Importance of individual elements within each component of physical literacy.

Component	Element	Combined importance (%)
Affective	Confidence	97.4
	Enjoyment	98.4
	Motivation	98
	Resilience	92.7
	Self-esteem	96.3
	Self-perception of competence	92.8
	Self-regulation - emotions	88
	Self-regulation - physical	91.7
	Value movement, sport, and physical activity	93.8
Cognitive	Identify and describe movement	64.3
	Reflect and improve own performance, including optimal challenges	84.5
	Creativity and imagination in application of movement	79.3
	Knowledge and understanding of the effects of movement, sport, and physical activity on the body	86.6
	Knowledge and understanding of the importance and benefits of movement, sport and physical activity	92.2
	Knowledge and understanding of the opportunities for movement, sport and physical activity	92.7
	Knowledge and understanding of safety and risk for self and others in movement, sport and physical activity contexts	84
	Knowledge and understanding of tactics, rules, and strategy	63.2
	Perceptual awareness	73.5
Physical	Agility	75.6
	Cardiovascular fitness	82.4
	Coordination	87
	Creativity in movement, sport and physical activity situations and contexts	76.7
	Fine motor skills	79.8
	Flexibility	77.7
	Functional movement skills	78.8
	Movement competence in different environments	64.8
	Movement skills	88
	Muscular endurance	71.5
	Power	63.8
	Reaction time	63.7
	Speed	64.8
	Strength	68.9
Social, moral, cultural	Ethics and morals (fairness and justice, inclusion, equity, integrity and respect)	86.5
	Relationships (building and maintaining relationships that enable a person to interact effectively with others)	95.4
	Society and culture (appreciation of cultural values which exist within groups, organisations and communities)	85
	Social skills (collaboration, communication, cooperation, leadership and conflict resolution)	94.3

#### 2.2.3 Knowledge of the term “physical literacy”

**Figure 1:**
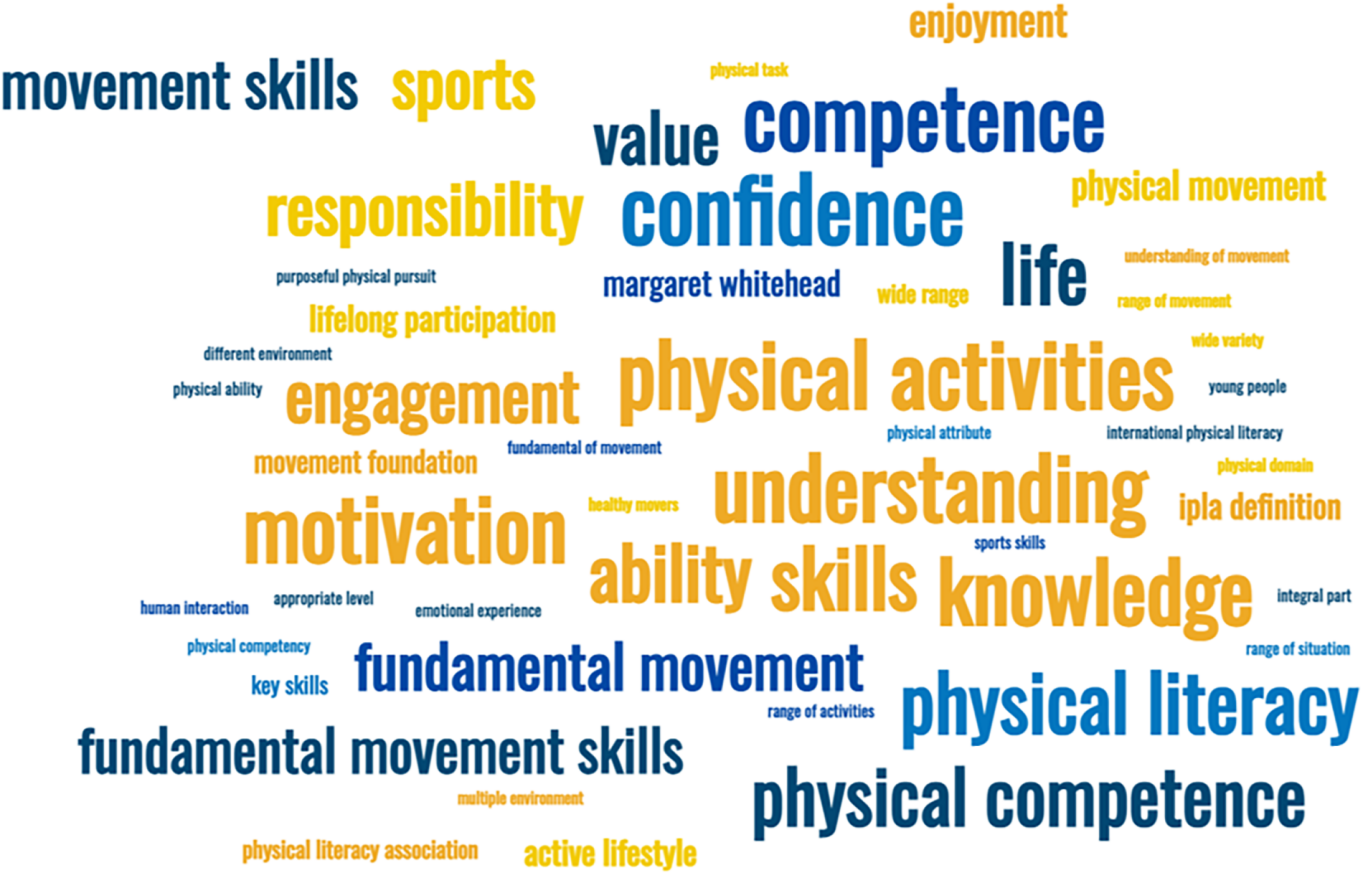
Word cloud as a visual representation of responses given by stakeholder to explain the term "physical literacy" (larger words reflect greater frequency in the free text responses provided by stakeholders).

In regard to the question “Have you heard of the term “Physical Literacy” before?”, by far the majority (90.2%, *n* = 174) responded that they had and 7.8% (*n* = 15) reported that they had not [with the remainder stating, “prefer not to say” (2.1%, *n* = 4)]. Those stakeholders that had heard of the term were asked how they would explain it, and overall, the participants demonstrated a grasp of the construct or at least parts of the construct, which referred to commonly conceived components of physical literacy. Approximately one in five respondents had a limited grasp of the concept. This was signified by stakeholders responding with the term “physical literacy” itself to describe physical literacy or simply replying “physical literacy association”, stating, “Margaret Whitehead” or “human interaction”. Figure 1 presents a word cloud as a visual representation of responses, where larger words reflect greater frequency in the free text responses provided by stakeholders.

The term “Physical Activity/Activities” was used by 78 respondents, often considered as the outcome of physical literacy and used in conjunction with some statements relating to some of the perceived components of physical activity. For example, “The motivation, confidence, physical competence, knowledge and understanding to take part in physical activity” occurred on 28 occasions as a singular phrase.

Singular components of physical literacy were also stated in combination - but not always all terms at the same time (confidence *n* = 90, understanding *n* = 75, motivation *n* = 73, knowledge *n* = 71, competence *n* = 69, movement *n* = 57, physical activity *n* = 42). Both “movement” and “competence” are considered under a heading of “physical competence”, this was by far the most stated component. Enjoyment was only mentioned seven times in stakeholder responses. Furthermore, statements relating to “for life” or “across the life course” were missing from over half the definitions, suggesting a general absence of physical literacy as a life course construct in the way respondents conceptualise physical literacy.

There were also examples where fundamental movement skills (*n* = 18; 10%) were cited as the definition of physical literacy. The IPLA [International Physical Literacy Association] was referred to (*n* = 10) in some cases as a sole response when citing the stakeholder's definition of physical literacy.

When asked if they were aware of any principles or philosophical assumptions of physical literacy, the answers were mixed, with a third (33.2%, *n* = 64) responding “yes”, a third (32.6%, *n* = 63) responding “no” and nearly a third (29%, *n* = 56) responding “don't know” and 5.2% (*n* = 10) preferring not to say. Collectively, this represents two-thirds of respondents who did not know of any principles or philosophies related to physical literacy. When asked to state what philosophies or principles exist, the subsequent responses were highly varied and illustrate that some respondents were aware of particular philosophies or principles, such as “existentialism” (*n* = 11), “phenomenology” (*n* = 11), and “monism” (*n* = 13). A number of responses were broader, referencing components (e.g., motivation, confidence, competence) of physical literacy, either in isolation or in combination, but often not in a complete form from any recognised definition of the term.

#### 2.2.4 Existing definitions of physical literacy

Stakeholders were then presented with four internationally recognised definitions of physical literacy; those used by IPLA (28), SHAPE America (29), Sport Australia (30), and Sport New Zealand (31), (See Table 3). Stakeholders were then asked a series of questions, responding via a 7-point Likert scale ranging on 5 points from Strongly Disagree to Strongly Agree and response options “Don't Know” or “Prefer not to say”.

**Table 3:** Internationally recognised definitions of physical literacy.

IPLA (2017)	SHAPE America (2019)
"Physical literacy can be described as the motivation, confidence, physical competence, knowledge and understanding to value and take responsibility for engagement in physical activities for life."	"Physical literacy is the ability to move with competence and confidence in a wide variety of physical activities in multiple environments that benefit the healthy development of the whole person."
Sport Australia (2019)	Sport New Zealand (2019)
"Physical literacy is lifelong holistic learning acquired and applied in movement and physical activity contexts. It reflects ongoing changes integrating physical, psychological, social, and cognitive capabilities. It is vital in helping us lead healthy and fulfilling lives through movement and physical activity. A physically literate person is able to draw on their integrated physical, psychological, social and cognitive capabilities to support health promoting and fulfilling movement and physical activity – relative to their situation and context – throughout the lifespan."	"A person's Physical Literacy is a combination of their motivation, confidence and competence to be active, along with their knowledge and understanding of how being active contributes to their life. Everyone has their own unique Physical Literacy that contributes to their overall wellbeing. It affects how, why and if they participate in physical activity throughout their life. It is important to note that a person's Physical Literacy reflects their context, environment, culture and world and physical literacy is a holistic concept, involving physical, social, emotional, cognitive and spiritual dimensions."

When asked if the definition fully captures the concept and key principles of physical literacy, the Sport New Zealand definition had the most respondents replying, “Strongly Agree” (51.8%, *n* = 100) and collectively replying either “Strongly Agree” or “Agree” (88.1%, *n* = 170) compared to the other definitions (See Table 4).

**Table 4:** Stakeholder rating for different definitions of physical literacy as to their capture of the concept and key principles of the term literacy as to their capture of the concept and key principles of the term.

	Strongly disagree	Disagree	Neutral	Agree	Strongly agree	Don't know	Prefer not to say
	*n* (%)	*n* (%)	*n* (%)	*n* (%)	*n* (%)	*n* (%)	*n* (%)
IPLA (2017)	3 (1.6)	15 (17.8)	23 (13)	76 (39.4)	69 (35.8)	4 (2.1)	1 (0.5)
SHAPE America (2015)	4 (2.1)	29 (15)	32 (16.6)	82 (42.5)	41 (21.2)	4 (2.1)	1 (0.5
Sport Australia (2019)	4 (2.1)	4 (2.1)	20 (10.4)	87 (45.1)	73 (37.8)	4 (2.1)	1 (0.5)
Sport New Zealand (2019)	2 (1)	5 (2.6)	11 (5.7)	70 (36.3)	100 (51.8)	4 (2.1)	1 (0.5)

When asked to rate if the definition was easy to understand (see Table 5), the IPLA (2017) definition had the largest proportion of responses as “Strongly Agree” (44%, *n* = 85), however, one in five respondents did not agree that the IPLA definition was easy to understand. The majority of respondents found the Sport Australia definition hard to understand.

**Table 5:** Stakeholder rating for different definitions of physical literacy as to its ease of understanding.

	Strongly disagree	Disagree	Neutral	Agree	Strongly agree	Don't know	Prefer not to say
	*n* (%)	*n* (%)	*n* (%)	*n* (%)	*n* (%)	*n* (%)	*n* (%)
IPLA (2017)	3 (1.6)	9 (4.7)	30 (15.5)	63 (32.6)	85 (44)	2 (1)	1 (0.5)
SHAPE America (2015)	3 (1.6)	18 (9.3)	41 (21.2)	74 (38.3)	54 (28)	2 (1)	1 (0.5)
Sport Australia (2019)	6 (3.1)	45 (23.3)	56 (29.0)	58 (30.1)	25 (13)	2 (1)	1 (0.5)
Sport New Zealand (2019)	4 (2.1)	30 (15.5)	39 (20.2)	70 (36.3)	47 (24.4)	2 (1)	1 (0.5)

**Figure 2:**
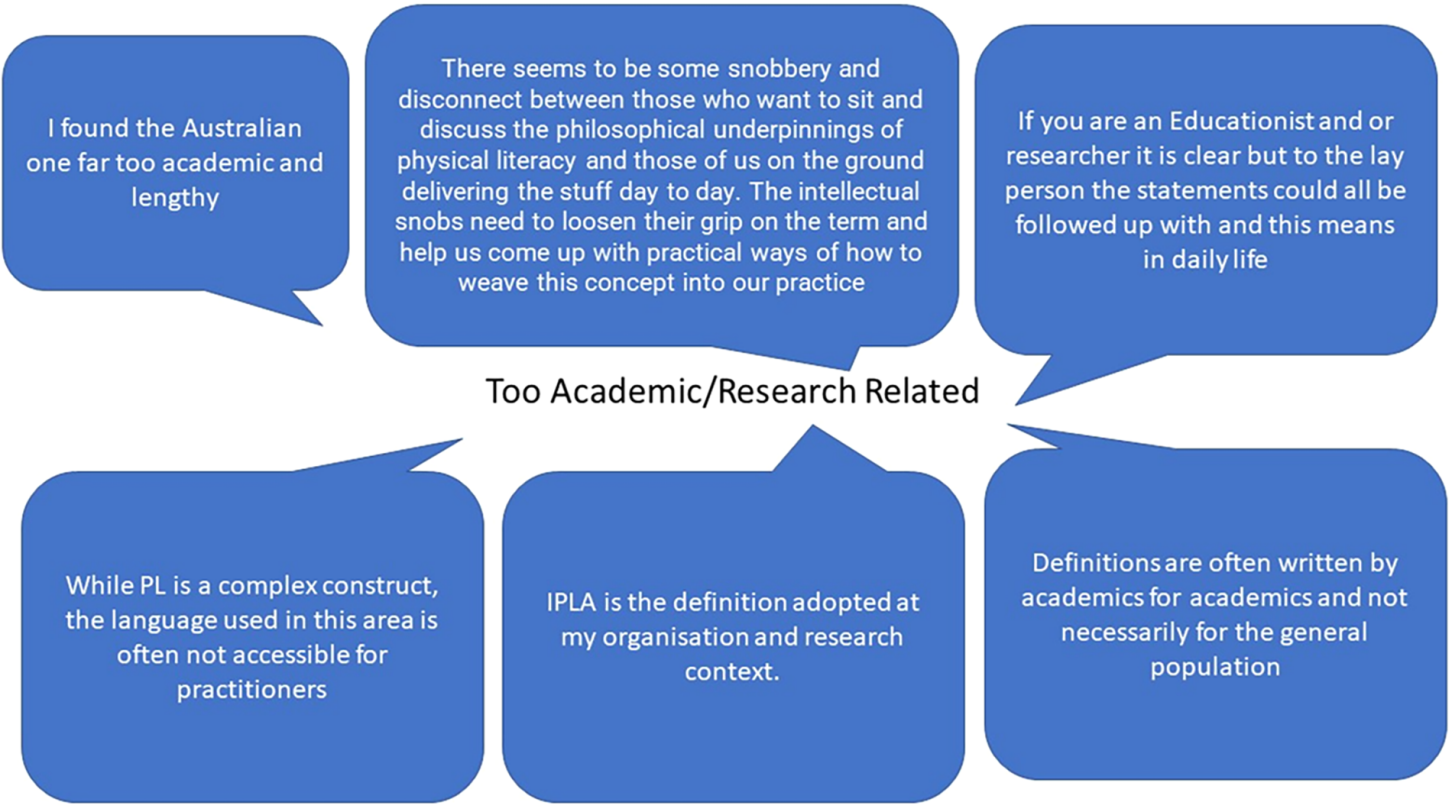
Pen profile responses related to the theme of academic/research related.

**Figure 3:**
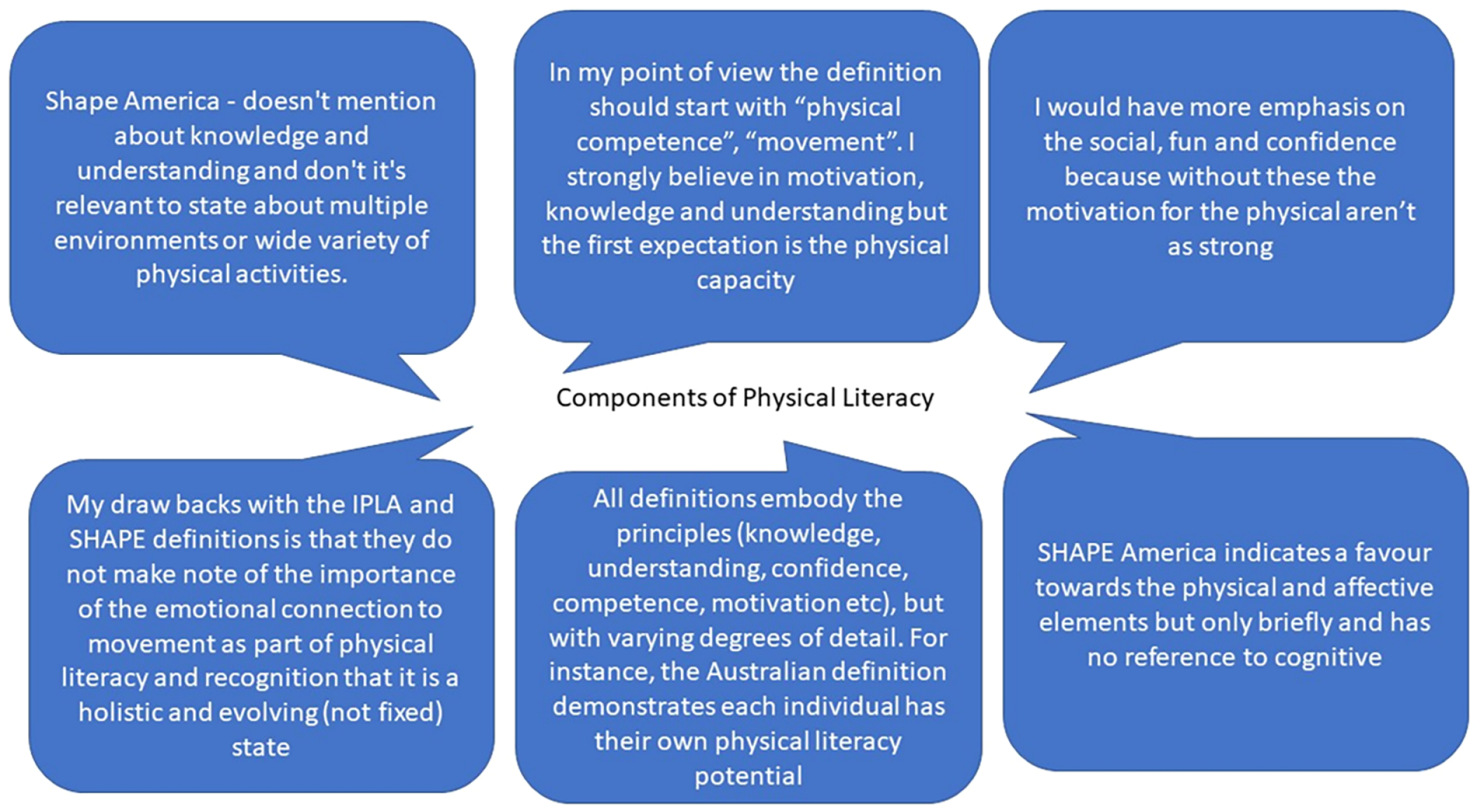
Pen profile responses related to the theme of components of physical literacy.

Stakeholders were then given the opportunity to expand, in free text form, regarding the definitions of physical literacy. Response data (*n* = 43) was broadly categorised into the following two themes: (1) Academic/Research related or (2) Components of Physical Literacy. Pen profiles of these responses are presented in Figures 2, 3 respectively.

A quarter of stakeholders commented that the definitions appeared too wordy, too academic and did not apply as well to practitioners. Indeed, positive responses were often aligned with concise and direct definitions - the length of the consensus statement and language used appeared to be important. A larger number of comments, and the main theme from these responses, related to discussion about the different components of physical literacy and how they might (or might not) feature in the four definitions stakeholders were presented with. Of the four definitions that were presented, the SHAPE (*n* = 1) and the Sport Australia (*n* = 7) definitions received the fewest positive comments in free text response. The Sport New Zealand definition (*n* = 14) had the greatest frequency of positive responses, followed by the IPLA (*n* = 10) definition.

#### 2.2.5 Value of physical literacy, training and resources

**Figure 4:**
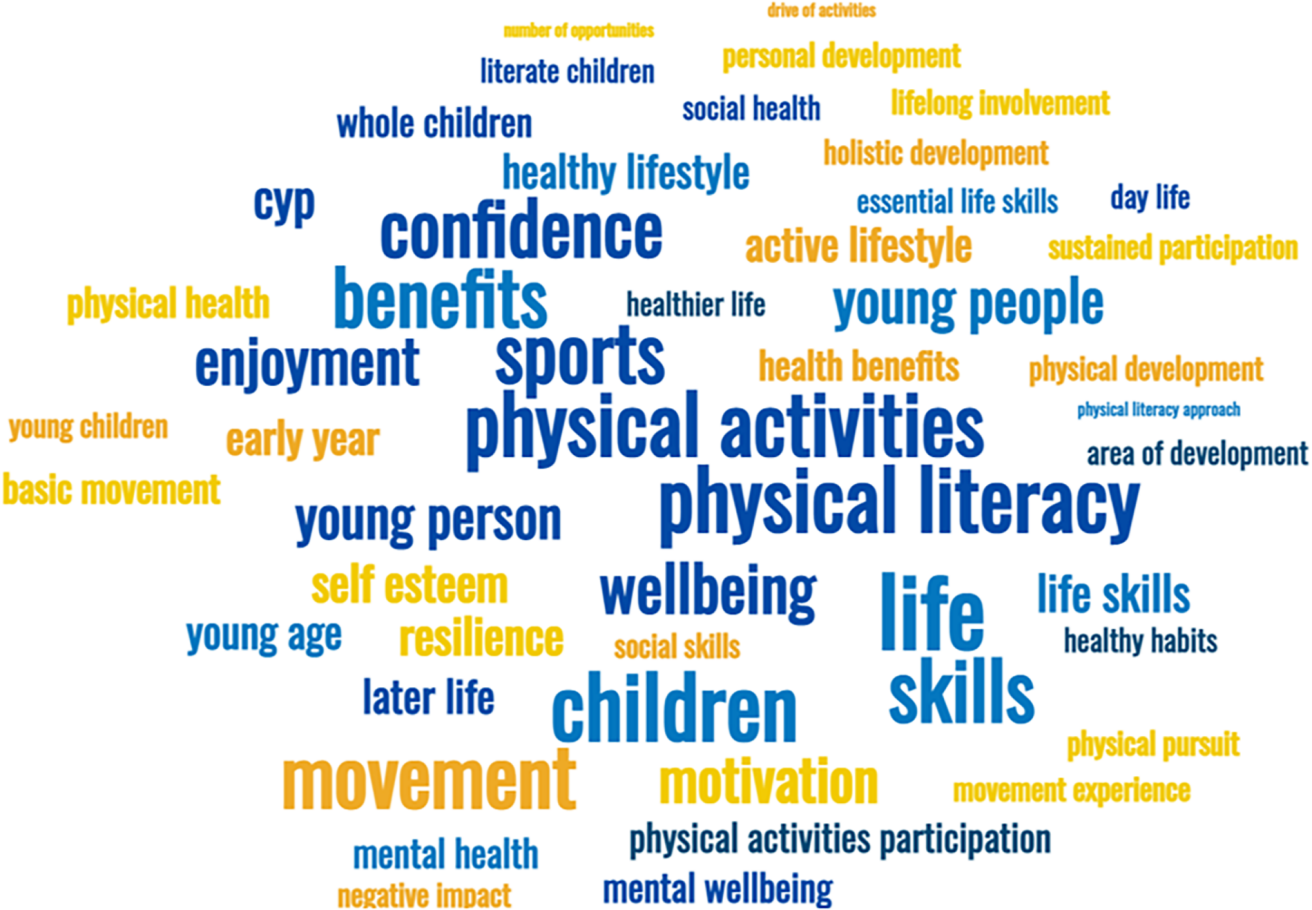
Word cloud as a visual representation of responses given by stakeholder to explain why physical literacy is important and what the benefits are (larger words reflect greater frequency in the free text responses provided by stakeholders).

Stakeholders were also asked to respond, using a scale of 0 (Not important) to 10 (Essential), to the question “How important is supporting physical literacy in young people?”. By far the majority (*n* = 162, 83.9%) responded with a score of 10/10 “essential”, and only three stakeholders (1.5%) responded with a score below 6/10 for this question. When asked a follow up free text question of why physical literacy is important and what the benefits are, there were frequent responses identifying that physical literacy was important for “health”/ to be healthy' (*n* = 50) or had some form of “benefit” for children (*n* = 20). The most frequently cited benefits were relating to “physical activity/activities” (*n* = 65), the development of “movement skills” (*n* = 27) and physical literacy as being related to participation in “sports” (*n* = 24). A word cloud representing responses from participants is presented in Figure 4.

#### 2.2.6 Opportunities arising from a shared definition of physical literacy

**Figure 5:**
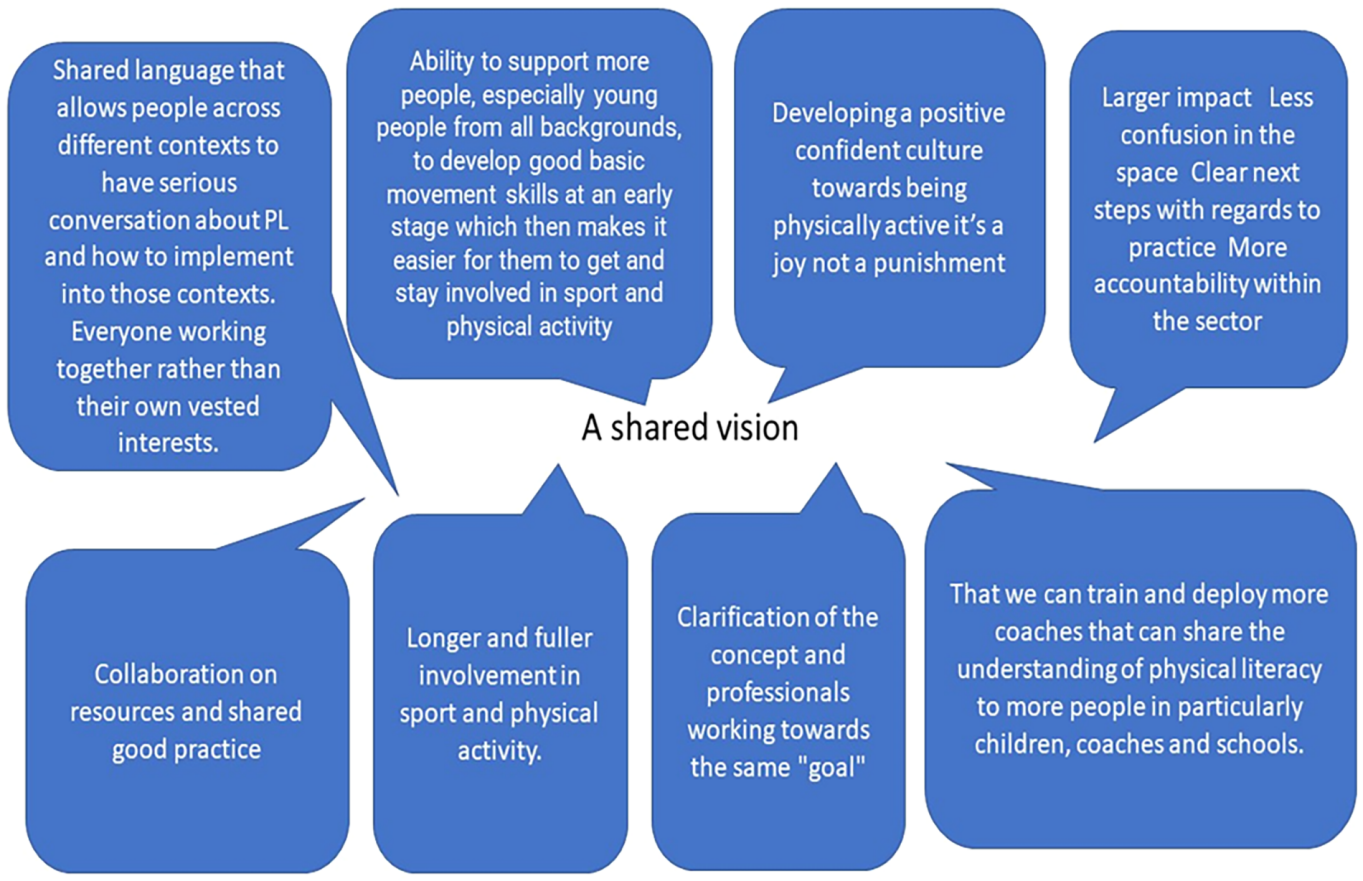
Pen profiles from stakeholders of what opportunities might arise from a shared understanding and vision of physical literacy.

The stakeholders then responded to a question asking what opportunities might arise from a shared understanding and vision of physical literacy. The responses to this question were overwhelmingly positive but varied. Responses broadly fitted into themes relating to an ability to have a positive impact/effect on those they worked with and/or collaborative opportunities across sectors/different job roles. Figure 5 presents pen profiles of the different types of responses given by stakeholders.

## 3 Discussion

The current study presents an understanding of the concept of physical literacy from the perspective of key multi-stakeholders in England working with and on physical literacy. Stakeholders play a pivotal role in translating research into practice but surprisingly their views have rarely been incorporated into the concept and evolution of physical literacy (21, 22, 24) and as yet multi-stakeholder views have not been presented collectively. The results of the present study therefore represent a unique insight on physical literacy from the perspective of stakeholders in England, which to date has not been forthcoming in the literature.

Stakeholders who responded to this survey provided a range of responses in relation to the concept of physical literacy. Implicit in the responses appeared to be a general valuing for physical literacy as a concept from the respondents. Thus, it is highly likely that those stakeholders most interested in, invested in, and involved in physical literacy in their organisations/work completed the survey. This work therefore provides a snapshot from that population, and there are likely a much wider range of stakeholders, where physical literacy may be relevant to their work that are not represented here.

The results of this stakeholder survey suggest that there remains considerable confusion in use of the term physical literacy in practice and confusion regarding the definition of physical literacy. Some stakeholders expressed a preference for a simple definition, others a more comprehensive definition. Most respondents offered surface level understandings of physical literacy, as indicated by the high level of abstraction in Young et al.'s concept analysis paper (see the Figure in the table). For example, only noting attributes from any given two domains, rather than including all domains, which are consistently used in physical literacy research. It is important to highlight, that in responses, inclusion of physical activity was missing from around one third of definitions, despite lifelong physical activity being the goal of physical literacy (32), and physical literacy being considered as an antecedent of physical activity (14).

Stakeholders considered the affective, social, physical and cognitive areas (domains) of learning to be most important for developing a person's positive relationship with movement, sport and physical activity for life. When specific elements of learning were examined for importance within each of these domains (e.g., cognitive domain - knowledge and understanding of the benefits of physical activity), there were a larger proportion of elements within the affective domain that were considered important. This observation aligns with findings from Belton et al. (21) who reported that Irish stakeholders felt the affective and cognitive domains were the most important in relation to physical literacy. In the present study, elements in the physical domain were rated as less important in general relative to the elements within the other domains. Most respondents suggested they were involved in physical literacy related activity and understood the term. However, when probed it appears that the physical literacy related activity they referred to was likely not specifically physical literacy related work, as their understanding of the term was inconsistent, often aligned to a particular element of physical literacy, ignoring key aspects of physical literacy as a construct. Moreover, there was a paucity of in-depth understanding of physical literacy provided by stakeholders with limited knowledge of key principles around the concept, particularly those related to physical literacy being inclusive, lifelong, a journey and being holistic as a concept. Such observations from the stakeholder group in the current study suggest a superficial understanding of physical literacy and one which ignores some of the key philosophical underpinnings of the concept (6).

It is also important to consider the results of the present study in relation to the domains and elements aspect of the survey. Firstly, most of the domains were considered as important, congruent with work in an Irish context (21). When considered in order of importance, affective, social and cognitive domains were most important with the physical domain ranked last, but paradoxically, the physical domain was the most commonly used term. The social domain was particularly valued as important (30) while the spiritual domain was not valued, unlike definitions from other countries (31), potentially highlighting cultural differences in physical literacy interpretation in different contexts. In respect to the different elements, 28 of 37 elements were rated as somewhat or extremely important, similar to prior work with Irish stakeholders (21). Given that stakeholders have offered a wide variety of elements (e.g., motivation, competence, etc.) that are important for development of physical literacy, a consensus statement definition may need to focus on domains rather than elements.

By providing evidence from stakeholders, this study provides an important evidence base which may support the development of future interventions targeting physical literacy, or tailored training to meet key stakeholders needs. Without addressing the aims of the current study, i.e., (i) capturing current understanding and perceptions of physical literacy among key stakeholders; and ii) identifying the challenges, needs and opportunities for supporting physical literacy among individuals/organisations in England, sustainably furthering the concept of physical literacy would not be possible as stakeholders are key in ensuring research translates to practice. Indeed, by collectively presenting the perspective of multi stakeholder groups, the current study seeks to begin a consensus process for the term physical literacy and offset the potential for multiple physical literacies which currently exists in academic discourse on the topic, as recently proposed by Young et al. (13). In practice, a multiversal construction of physical literacies (13) may cause more confusion and less effectiveness in coaching and educational settings when teachers, coaches, sports providers, NGBs, health professionals are working to foster physical literacy in the communities they work with.

## 4 Strengths and limitations

The strengths of the current study are, the comprehensive nature of the survey, and subsequent views gained reflecting multiple aspects of physical literacy, unlike prior research which has tended to focus on specific aspects of physical literacy [e.g., assessment of physical literacy, as in Goss, et al. (22)]. Secondly, gaining the views of multi-sectoral stakeholder groups is a key element of the present study, where prior work has tended to focus on stakeholders from single sectors (21, 22) or has focused solely on academic experts (3). Providing this multi-sectoral viewpoint is essential in creating a shared understanding and buy-in from all stakeholder sectors. While physical literacy has tended to be front and centre within an educational context, other sectors and stakeholder groups need to feel represented and have opportunity to coproduce a definition and future activity related to physical literacy. That said, the study is not without limitations. Participants were restricted to stakeholders in England who responded to an open call to participate in the survey and the data presented may be more reflective of the structure and cultural context in which physical literacy is positioned in England. We also note that the total number of participants is relatively modest in comparison say to the total number of teachers in England, and a greater number of respondents would have extended the conclusions that could be drawn from this work. It is however important to note that there was a broad spread of stakeholder roles and sectors represented. It would be useful for future work to examine differences in perceptions and practice of physical literacy in stakeholders from different countries and cultural contexts. This was, however, beyond the remit of the present study. As a consequence of our recruitment strategy, it is also likely that stakeholders who were more interested in physical literacy were more likely to participate. Furthermore, while the data presents a snapshot of perceptions of physical literacy from stakeholders in England, future in-depth qualitative insights would be useful in unpacking how strategies to develop physical literacy might best be embedded into practice. Such an approach would also be useful in establishing a shared definition of what the term “physical literacy” comprises for stakeholders working in and around the topic. We are aware of the descriptive nature of the approach used in the current study. This approach is deliberate and useful as no prior work had examined the perceptions and practices of stakeholders concerning physical literacy.

## 5 Conclusion

Key implications arise from the present study. While stakeholders are aware of the term “physical literacy” and hold value of it within their practice, there remain key misconceptions relating to what physical literacy is, and debate as to whether any existing definitions truly capture the construct of physical literacy. Providing evidence-based resources (e.g., webinars, infographics, guidebooks) for stakeholders to reach consensus regarding what physical literacy is, and what it is not, would be a useful next step. Once established, relevant national bodies (e.g., Sport England), with responsibility for creating positive trajectories of health through physical activity, could look to develop guidance, professional development, or training to help stakeholders embed physical literacy in their work.

## Data Availability

The raw data supporting the conclusions of this article will be made available by the authors, without undue reservation.
