# Supplementary material for: Stakeholder perceptions of physical literacy: results from a national consultation in England
Source: Front Sports Act Living. 2024 Oct 17;6:1457845. doi: 10.3389/fspor.2024.1457845 (PMC11524871; doi:10.3389/fspor.2024.1457845)
Supplement: Supplementary file 1 [file Datasheet1.pdf]

Sport England Physical Literacy Stakeholder Engagement Survey

Page 1: Participant Information

We would like to invite you to take part in a research study. Before you decide you need to understand why the research is being conducted and what it would involve for you. Please take time to read the following information carefully. If you have any questions, if anything is not clear, or you would like more information, then our contact details are provided below. Take time to decide whether or not you wish to take part.

What is the research study about?

Researchers from Coventry University and Liverpool John Moores University are interested in the current understanding and perceptions of physical literacy among the workforce and organisations/employers within the physical activity, sport, education, health, child, voluntary and community sectors. We want to learn about your understanding of physical literacy and what matters in helping children and young people to have a positive relationship with movement, sport and physical activity for life. This is part of a research study commissioned by Sport England aiming to:

- Capture current understanding and perceptions of physical literacy among key stakeholders
- Identify the challenges, needs and opportunities for supporting physical literacy among individuals/organisations
- Create a shared vision and understanding for physical literacy in England

Why have I been invited?

Because you are a stakeholder or organisational partner working with children and young people in the physical activity, sport, education, health, child, voluntary and community sectors.

Do I have to take part?

Taking part in this study is entirely voluntary, it is up to you to decide. We will describe the study and then ask you to click to confirm that you are happy to participate and you have agreed to take part. You are free to withdraw at any time, without giving a reason.

What will happen to me if I take part?

The study comprises a 20 minute online survey and responses are completed anonymously. You can complete the survey as an individual or on behalf of an organisation. We ask you to complete the survey in your own time and we genuinely value your input.

Withdrawal

You can withdraw at any point until the survey is completed. After this point we will not be able to withdraw your data due to the fact that the survey is anonymous.

What are the benefits?

We cannot promise the study will help you personally. The study is focused on better understanding the needs of stakeholders to enable Sport England to better support individuals and organisations to provide experiences that foster a positive relationship wth movement, sport and physical activity among children and young people.

What are the risks?

There are no risks involved in taking part in this project.

What happens to the information?

Procedures for handling, processing, storage and destruction of their data match the Caldicott principles, the Data Protection Act 1998 and the EU General Data Protection Regulations.

All data gathered will only be used for the purposes described above and only the principal researchers involved (Prof Mike Duncan, Dr Lawrence Foweather) will have access to the data. We will store the data on a University secure server. Responses to the survey will be analysed and we intend to publish anonymised aggregated data ('no names' group level findings) in a report that will be shared with Sport England. We may make this report publicly available on the University and Sport England websites. The anonymous findings will also be shared with the Physical Literacy Expert Panel - around 40 individuals comprised of researchers and stakeholders - and may be published in a scientific journal article.

Who has reviewed this study?

This study has been reviewed and approved by the Ethics Committee at Coventry University and the procedures in place in the study adhere to the Code of Conduct of the British Association of Sport and Exercise Sciences

What if I have more questions?

If you have any further questions please get in touch with the principal researcher for the study, Prof. Mike Duncan, School of Life Sciences, Science and Health Building, Coventry, CV1 5FB, Telephone: 024776888613 or e-mail: michael.duncan@coventry.ac.uk. Alternatively you can contact Prof Richard Dashwood, Associate Pro-Vice Chancellor Research: 02477659725 or email: ab8677@coventry.ac.uk

---

I confirm that I have read and understood the participant information.

- ○ Yes
- ○ No

I understand that my participation is voluntary and that I am free to withdraw at anytime without giving a reason until my responses are submitted, at which point my responses become anonymous.

- ○ Yes
- ○ No

I understand that all data will be processed and stored anonymously and used for research purposes. This data will be stored on the university network.

- ○ Yes
- ○ No

I agree to take part in this study

- ○ Yes
- ○ No

Page 2: Section 1. INDIVIDUAL ROLE AND ORGANISATION

Are you submitting these responses in a personal capacity or on behalf of an organisation?

- [ ] Personal Capacity
- [ ] Organisation

What is the name of your organisation? (*optional - please note, your comments will be presented anonymously with no mention of your role/affiliation)*

What is your employment status as it relates to this questionnaire?

- Employed - full time
- Employed - part time
- Self-employed
- Combined full/part time and self-employed
- Unemployed
- Volunteer
- Retired
- Prefer not to say

What is your job title as it relates to this questionnaire?

- Allied health professional
- Coach
- Coach developer / educator
- College lecturer
- Director / Owner
- Director of public health
- Doctors
- Group exercise instructor
- Professional development
- Health and social care professional
- Health practitioner
- Health trainer
- Health visitor

- Leisure / fitness centre manager
- Manager
- Personal trainer
- Physiotherapist
- Play worker
- Psychologist
- Public health consultant
- Programme manager / lead
- Pre-school teacher
- Primary school teacher
- Teaching assistant
- Secondary school (non-PE) teacher
- Secondary school PE teacher (specialist)
- School principal / Headteacher
- Scientist / Researcher
- Sports leader
- Safeguarding officer
- Sports / Physical activity coordinator
- Sports development officer
- Sports therapist
- Student
- Swimming teacher
- University lecturer
- Volunteer
- Youth worker
- Yoga or pilates instructor
- Prefer not to say
- Other (please specify)

If 'Other' please specify

With what sector(s) do you associate? (*tick all that apply*)

- Childcare
- Community
- Education
- Fitness and Leisure
- Government
- Healthcare

- Physical Activity
- Public Health
- Recreation
- Research
- Sport
- Voluntary
- Prefer not to say
- Other (please specify)

if 'Other' please specify

Do you/your organisation target, are restricted to, or intend to benefit people living in a particular geographical area? (*tick all that apply*)

- National
- East Midlands
- East of England
- London
- North East
- North West
- South East
- South West
- Yorkshire and The Humber
- West Midlands
- Not applicable

Who are the target audiences for you/your organisation? (*tick all that apply*)

- Young children (under 5s)
- Children (5-11 years)
- Young people (12-17 years)
- Adults (18-64 years)
- Older adults (65 and over)
- Ethnically diverse communities
- Communities experiencing racial inequity
- Faith communities
- Gender – males and boys

- [ ] Gender – women and girls
- [ ] Disabled persons
- [ ] LGBTQ+
- [ ] People who are educationally or economically disadvantaged
- [ ] Health conditions
- [ ] Migrants
- [ ] Other (please specify)

If 'Other' please specify

In this section, we want to learn what you think matters in helping children and young people to have a positive relationship with movement, sport and physical activity for life.

Page 4

How important are the following areas of learning in relation to developing a person's positive relationship with movement, sport and physical activity for life? ✱ *Required*

	Not important at all	Somewhat unimportant	Neutral	Somewhat important	Extremely important	Don't know	Prefer not to say
AFFECTIVE (emotional or psychological aspects: feelings, values, emotions, and attitudes towards movement, sport and physical activity)	☐	☐	☐	☐	☐	☐	☐
COGNITIVE (knowledge and understanding required for movement, sport and physical activity)	☐	☐	☐	☐	☐	☐	☐
CREATIVITY (imagination, invention, flexible thinking, expression, originality in movement, sport and physical activity contexts)	☐	☐	☐	☐	☐	☐	☐
CULTURAL (understanding, valuing, and appreciating cultural diversity in movement, sport and physical activity contexts; awareness of cultural norms; challenging discrimination)	☐	☐	☐	☐	☐	☐	☐
LANGUAGE (spoken and written communication/language, vocabulary, literacy)	☐	☐	☐	☐	☐	☐	☐
MORAL (considered choices around behaviours and values; development of moral attributes or virtues)	☐	☐	☐	☐	☐	☐	☐
PHYSICAL (movement skills, fitness, body control and coordination required for movement, sport and physical activity)	☐	☐	☐	☐	☐	☐	☐
SENSORY (sight, touch, smell, hearing, taste, vestibular and proprioception)	☐	☐	☐	☐	☐	☐	☐

SOCIAL (social skills to effectively interact with others in movement, sport and physical activity contexts)	☐	☐	☐	☐	☐	☐	☐
SPIRITUAL (guiding beliefs, principles or values that help give meaning, purpose, and direction to a person’s life)	☐	☐	☐	☐	☐	☐	☐

With regards to AFFECTIVE development (*psychological and emotional capabilities*), how important are the following elements of learning in relation to developing a person's positive relationship with movement, sport, and physical activity for life?

	Not important at all	Somewhat unimportant	Neutral	Somewhat important	Extremely important	Don't know	Prefer not to say
Confidence	☐	☐	☐	☐	☐	☐	☐
Enjoyment	☐	☐	☐	☐	☐	☐	☐
Motivation	☐	☐	☐	☐	☐	☐	☐
Resilience	☐	☐	☐	☐	☐	☐	☐
Self-esteem	☐	☐	☐	☐	☐	☐	☐
Self-perception of competence in relation to movement, sport and physical activity	☐	☐	☐	☐	☐	☐	☐
Self-regulation - emotions (ability to manage emotions and feelings in relation to movement, sport and physical activity)	☐	☐	☐	☐	☐	☐	☐
Self-regulation - physical (ability to recognise and manage physical signals such as pain, fatigue and exertion)	☐	☐	☐	☐	☐	☐	☐
Value movement, sport, and physical activity	☐	☐	☐	☐	☐	☐	☐

Are there any other AFFECTIVE elements of learning missing from the list above that you consider to be important in developing a person's positive relationship with movement, sport, and physical activity for life? *Optional*

With regards to COGNITIVE development, how important are the following elements of learning in relation to developing a person's positive relationship with movement, sport, and physical activity for life?

	Not important at all	Somewhat unimportant	Neutral	Somewhat important	Extremely important	Don't know	Prefer not to say
Ability to identify and describe movement	☐	☐	☐	☐	☐	☐	☐
Ability to reflect and improve own performance, including setting optimal challenges	☐	☐	☐	☐	☐	☐	☐
Creativity and imagination in application of movement	☐	☐	☐	☐	☐	☐	☐
Knowledge and understanding of the effects of movement, sport, and physical activity on the body	☐	☐	☐	☐	☐	☐	☐
Knowledge and understanding of the importance and benefits of movement, sport and physical activity	☐	☐	☐	☐	☐	☐	☐
Knowledge and understanding of the opportunities for movement, sport and physical activity	☐	☐	☐	☐	☐	☐	☐
Knowledge and understanding of safety and risk for self and others in movement, sport and physical activity contexts	☐	☐	☐	☐	☐	☐	☐
Knowledge and understanding of tactics, rules, and strategy	☐	☐	☐	☐	☐	☐	☐
Perceptual awareness (tacit knowledge used to quickly recognise the environment and make accurate decisions based on experiences, observations, emotions, and intuition)	☐	☐	☐	☐	☐	☐	☐

Are there any COGNITIVE elements of learning missing from the list above that you consider to be important in developing a person's positive relationship with movement, sport, and physical activity for life? *Optional*

With regards to PHYSICAL development, how important are the following elements of learning in relation to developing a person’s positive relationship with movement, sport, and physical activity for life?

	Not important at all	Somewhat unimportant	Neutral	Somewhat important	Extremely important	Don’t know	Prefer not to say
Agility	☐	☐	☐	☐	☐	☐	☐
Cardiovascular fitness	☐	☐	☐	☐	☐	☐	☐
Coordination	☐	☐	☐	☐	☐	☐	☐
Creativity in movement, sport and physical activity situations and contexts	☐	☐	☐	☐	☐	☐	☐
Fine motor skills (small muscles working with the brain and nervous system to control movements to do things like eating, writing, manipulating objects, getting dressed)	☐	☐	☐	☐	☐	☐	☐
Flexibility	☐	☐	☐	☐	☐	☐	☐
Functional movement skills (the ability to move with proper muscle and joint function, and strength, e.g., squat, lunge, and push-ups)	☐	☐	☐	☐	☐	☐	☐
Movement competence in different environments (e.g., land, water, ice, or snow)	☐	☐	☐	☐	☐	☐	☐
Movement skills (i.e., stability [e.g., balancing], locomotor [e.g., running], and object-manipulation [e.g., catching, kicking])	☐	☐	☐	☐	☐	☐	☐
Muscular endurance	☐	☐	☐	☐	☐	☐	☐
Power	☐	☐	☐	☐	☐	☐	☐
Reaction time	☐	☐	☐	☐	☐	☐	☐
Speed	☐	☐	☐	☐	☐	☐	☐
Strength	☐	☐	☐	☐	☐	☐	☐

Are there any PHYSICAL elements of learning missing from the list above that you consider to be important in developing a person’s positive relationship with movement, sport, and physical activity for life? *Optional*

With regards to SOCIAL, MORAL and CULTURAL development, how important are the following elements of learning in relation to developing a person’s positive relationship with movement, sport, and physical activity for life?

	Not important at all	Somewhat unimportant	Neutral	Somewhat important	Extremely important	Don’t know	Prefer not to say
Ethics and morals (fairness and justice, inclusion, equity, integrity and respect)	☐	☐	☐	☐	☐	☐	☐
Relationships (building and maintaining relationships that enable a person to interact effectively with others)	☐	☐	☐	☐	☐	☐	☐
Society and culture (appreciation of cultural values which exist within groups, organisations and communities)	☐	☐	☐	☐	☐	☐	☐
Social skills (collaboration, communication, cooperation, leadership and conflict resolution)	☐	☐	☐	☐	☐	☐	☐

Are there any SOCIAL, MORAL and CULTURAL elements of learning missing from the list above that you consider to be important in developing a person’s positive relationship with movement, sport, and physical activity for life?
Optional

Page 9

Have you heard of the term physical literacy before?

- Yes
- No
- Prefer not to say

If you have heard of the term, how would you define and explain physical literacy? (*please feel free to use your own words or text from an existing definition that you agree with*)

Are you aware of any principles or philosophical assumptions of physical literacy?

- Yes
- No
- Don't Know
- Prefer not to say

If 'Yes' please state what philosophies or principles

Where did you source this information? *Optional*

There are several international approaches to the definition of physical literacy. Some examples are below. There are subtle differences between each definition in terms of the areas of learning and development noted, and the depth of explanation of the concept offered. We want to learn which definition of physical literacy resonates with you/your organisation and why.

"Physical literacy can be described as the motivation, confidence, physical competence, knowledge and understanding to value and take responsibility for engagement in physical activities for life." (IPLA, 2017)

"Physical literacy is the ability to move with competence and confidence in a wide variety of physical activities in multiple environments that benefit the healthy development of the whole person." (SHAPE America, 2019)

"Physical literacy is lifelong holistic learning acquired and applied in movement and physical activity contexts. It reflects ongoing changes integrating physical, psychological, social, and cognitive capabilities. It is vital in helping us lead healthy and fulfilling lives through movement and physical activity. A physically literate person is able to draw on their integrated physical, psychological, social and cognitive capabilities to support health promoting and fulfilling movement and physical activity – relative to their situation and context – throughout the lifespan." (Sport Australia, 2019)

"A person’s Physical Literacy is a combination of their motivation, confidence and

competence to be active, along with their knowledge and understanding of how being active contributes to their life. Everyone has their own unique Physical Literacy that contributes to their overall wellbeing. It affects how, why and if they participate in physical activity throughout their life. It is important to note that a person’s Physical Literacy reflects their context, environment, culture and world and physical literacy is a holistic concept, involving physical, social, emotional, cognitive and spiritual dimensions." (Sport New Zealand, 2019)

The following questions are about you/your organisations perceptions of these definitions of physical literacy and their relevance to your current delivery or provision.

The definition is relevant and appropriate to the concept of physical literacy

	Strongly disagree	Disagree	Neutral	Agree	Strongly agree	Don't know	Prefer not to say
IPLA (2017)	☐	☐	☐	☐	☐	☐	☐
SHAPE America (2019)	☐	☐	☐	☐	☐	☐	☐
Sport Australia (Australian Sports Commission, 2019)	☐	☐	☐	☐	☐	☐	☐
Sport New Zealand (2019)	☐	☐	☐	☐	☐	☐	☐

The definition fully captures the concept and key principles of physical literacy

	Strongly disagree	Disagree	Neutral	Agree	Strongly agree	Don't know	Prefer not to say
IPLA (2017)	☐	☐	☐	☐	☐	☐	☐
SHAPE America (2019)	☐	☐	☐	☐	☐	☐	☐
Sport Australia (Australian Sports Commission, 2019)	☐	☐	☐	☐	☐	☐	☐
Sport New Zealand (2019)	☐	☐	☐	☐	☐	☐	☐

The definition is easy to understand and appropriately worded

	Strongly disagree	Disagree	Neutral	Agree	Strongly agree	Don't know	Prefer not to say
IPLA (2017)	☐	☐	☐	☐	☐	☐	☐
SHAPE America (2019)	☐	☐	☐	☐	☐	☐	☐
Sport Australia (Australian Sports Commission, 2019)	☐	☐	☐	☐	☐	☐	☐
Sport New Zealand (2019)	☐	☐	☐	☐	☐	☐	☐

If you wish, please expand upon your answers above and state which definition(s) does or does not resonate with you/your organisation, and why. *Optional*

Page 11

From your perspective, how important is supporting physical literacy in children and young people?

	1 (Not Important)	2	3	4	5	6	7	8	9	10 (Essential)
How important is supporting physical literacy in children and young people?	☐	☐	☐	☐	☐	☐	☐	☐	☐	☐

In your opinion, why is physical literacy important? What are the benefits?

Optional

Page 12: Section 4. EDUCATION, TRAINING, PROGRAMMES, TOOLS AND RESOURCES

This next section is about what physical literacy education and training, programmes, tools and resources currently exist in England. This might include supporting the areas of learning that are related to physical literacy and fostering a positive relationship with movement, sport and physical activity for life.

Are you aware of any education and training, programmes, tools and/or resources aimed at physical literacy, which you would like to share with us? Please also include any that you/your organisation offer as relevant.

Education and training

- Yes
- No
- Prefer not to say

If 'Yes' please describe which education and training (e.g., aims, target population, name of education/training/professional development course, provider, website links)

Programmes

- Yes
- No
- Prefer not to say

If 'Yes' please describe what programmes (e.g., aims, physical literacy related elements targeted, target audience,

number of participants, name of programme, provider, website links)

Tools and resources

- Yes
- No
- Prefer not to say

If 'Yes' Please describe what tools and resources (e.g., Titles, type of resource/products [website, media, books, videos, etc.], website links)

Do you use any tools and/or resources to develop physical literacy in your current practice?

- Yes
- No
- Prefer not to say

If 'Yes' which tools/resources do you use in your current practice

Page 13: Section 5. CHALLENGES, SUPPORT AND OPPORTUNITIES

How would you rate your level of expertise in the area of physical literacy?

	None	Minimal	Adequate	Good	Excellent
Level of expertise	☐	☐	☐	☐	☐

What are the top 3 challenges in developing physical literacy among your target audience(s)? *Optional*

What are the top 3 challenges to adopting, promoting and supporting physical literacy for you/your organisation? *Optional*

What are the top 3 practical things needed to help you / your organisation adopt, support, and promote physical literacy? *Optional*

Page 14: Section 6. FINAL THOUGHTS

What opportunities do you/your organisation see in the long-term arising from a shared understanding and vision of physical literacy in England? *Optional*

Are there any ideas or other thoughts which you would like to share? Please share anything else that you'd like to feedback to us. *Optional*

Please leave your email if you would be interested in contributing to the development of a physical literacy consensus statement for England and/or its adoption. This may involve the research team contacting you in the future in relation to the project as it evolves. *Optional*

Page 15: Section 7. OPTIONAL RESPONDENT DEMOGRAPHICS QUESTIONS

This section of the survey is completely optional. We ask the following questions surrounding protected characteristics in order to assess the representativeness and diversity of the survey respondants. These responses are completely confidential and will be not be linked to any of your responses. You can either choose not to complete this section or you can complete the section and skip any specific question you do not wish to answer by clicking 'prefer not to say'.

I am happy to provide information about my personal characteristics (e.g., age, gender, ethnicity, religion, sexuality and disability)

- YES
- NO

What is your gender?

- Male
- Female
- Non-binary
- Other
- Prefer not to say

What is your age?

Which of the following best describes your ethnic group or background?

- White British
- Other White background
- Mixed
- Asian or Asian British
- Black or Black British
- Other Ethnic Group
- Prefer not to say

Do you have any physical or mental health conditions or illnesses that have lasted or are expected to last 12 months or more?

- Yes
- No
- Prefer Not to Say

Do these physical or mental health conditions or illnesses have substantial effect on your ability to do normal daily activities?

- Yes
- No
- Prefer not to say

Does this disability or illness affect you in any of the following areas?

- Long term pain
- Chronic health condition
- Mobility
- Dexterity
- Mental health
- Visual
- Breathing
- Memory
- Hearing
- Learning
- Speech
- Behavioural
- Other
- None of these
- Prefer not to say

What is your religion, even if you are not currently practising?

- Christian (including Church of England, Catholic, Protestant and all other Christian denominations)
- Buddhist
- Hindu
- Jewish
- Muslim
- Sikh
- Any other religion
- No religion

- Don’t know
- Prefer not to say

Which of the following options best describes how you think of yourself?

- Heterosexual or straight
- Gay or lesbian
- Bisexual
- Other
- Don’t know

Page 16: Final page

This is the end of the survey. Thank you very much for your time and responses. Your thoughts and perceptions are greatly appreciated and highly valued. We will use the learning and insight gleaned from this survey to inform the development of a physical literacy consensus statement for England.
